# Prenatal exposure of a girl with autism spectrum disorder to 'horsetail' (Equisetum arvense) herbal remedy and alcohol: a case report

**DOI:** 10.1186/1752-1947-5-129

**Published:** 2011-03-31

**Authors:** Juan A Ortega García, Mario G Angulo, Elías J Sobrino-Najul, Offie P Soldin, Alberto Puche Mira, Eduardo Martínez-Salcedo, Luz Claudio

**Affiliations:** 1 Pediatric Environmental Health Specialty Unit, Neuropediatric Unit, Department of Pediatrics, University Hospital Virgen of Arrixaca, Murcia, Spain; 2 Network PregnaTox, Departments of Medicine, Oncology, Physiology, Obstetrics and Gynecology, Georgetown University Medical Center, Washington DC, USA; 3 Neuropediatric Section, Department of Pediatrics, University Hospital Virgen of Arrixaca, Murcia, Spain; 4 Division of International Health, Department of Preventive Medicine, Mount Sinai School of Medicine, New York, NY, USA

## Abstract

**Introduction:**

Autism is a complex neurodevelopmental disorder in which the interactions of genetic, epigenetic and environmental influences are thought to play a causal role. In humans, throughout embryonic and fetal life, brain development is exquisitely susceptible to injury caused by exposure to toxic chemicals present in the environment. Although the use of herbal supplements during pregnancy is relatively common, little information is available on their association with fetal neurodevelopment. This is, to the best of our knowledge, the first report in the literature to associate a new plausible mechanism of neurodevelopmental toxicity with a case of autism spectrum disorder through a vitamin deficiency potentiated by concomitant use of herbal supplements and ethanol exposure.

**Case presentation:**

We describe the pediatric environmental history of a three-year-old Caucasian girl with an autism spectrum disorder. We utilized her pediatric environmental history to evaluate constitutional, genetic, and environmental factors pertinent to manifestation of neurodevelopment disorders. Both parents reported prenatal exposure to several risk factors of interest. A year prior to conception the mother began a weight loss diet and ingested 1200 mg/day of 'horsetail' (*Equisetum arvense*) herbal remedies containing thiaminase, an enzyme that with long-term use can lead to vitamin deficiency. The mother reported a significant weight loss during the pregnancy and a deficiency of B-complex vitamins. Thiamine (vitamin B1) deficiency could have been potentiated by the horsetail's thiaminase activity and ethanol exposure during pregnancy. No other risk factors were identified.

**Conclusions:**

A detailed and careful pediatric environmental history, which includes daily intake, herbal remedies and ethanol exposure, should be obtained from all patients with autism spectrum disorder. Maternal consumption of ethanol and of herbal supplements with suspected or potential toxicity should be avoided during pregnancy. The prospective parents should perform preconception planning before pregnancy.

## Introduction

Autism refers to a set of neurodevelopmental disorders characterized by impaired social interaction, restricted communication, and repetitive, stereotypic behaviors. The number of children reported as having autism spectrum disorders (ASDs) has risen dramatically since the early 1990s. The prevalence of autism currently reported in developed countries is 3 to 7 cases per 1000 children [1-3]. Genetic predisposition could explain approximately 7% to 8% of all cases and that percentage is expected to increase with progress in autism research. Monozygotic twin concordance is found to be around 70% and 90% for autistic behavioral traits only. However, in dizygotic twins, it does not appear to be higher than in isolated brothers. Some genetic diseases such as fragile X syndrome, Down's syndrome, Angelman syndrome, Rett syndrome and Cohen syndrome significantly increase the risk of developing autism [4].

Despite the advances in autism research, certain clinical and epidemiological aspects of autism remain largely unknown. For example, the sporadic appearance and clinical heterogeneity in different members of a family diagnosed as autistic and others with 'autistic traits' suggest that autism is a complex neurodevelopmental disorder in which the interactions of genetic, epigenetic and environmental influences plays a causal role [5]. The pediatric environmental history (PEH) is a critical element in clinical documentation and it is used to register the absence or presence of risk factors (RFs) associated with the occurrence of diseases such as the ASD. The PEH employs a series of basic and concise questions including genetic, genealogical and constitutional aspects that allow the clinicians to identify environmental RFs associated with disease [6]. The purpose of this case report is to illustrate the role of PEH in identifying the RFs associated with ASD and to explain the potential relationship between horsetail exposure and ASD.

## Case presentation

We present the case of a three-year-old Caucasian girl with ASD, focusing on her PEH (Table 1). She was born after 41-weeks gestation via caesarean section and had an Apgar score of 9/10. A birth weight of 3.85 kg, head circumference of 37 cm and length at birth of 50 cm were recorded. No dyschromias or malformations were found. Our patient's growth and karyotype were otherwise normal. She was breastfed until the age of nine weeks. By five months, she was able to hold her head steady and erect, and was able to walk on her own at 15 months of age. Our patient currently walks on her tiptoes and began this when she first started walking. On examination, our patient had normal growth and anthropometric data. Her muscle tone was moderately decreased, widespread and symmetric, with normal osteotendinous nerve reflexes. Acquisition and delayed maturation of language was observed. Sensory perception and attention were thought to be normal until 12 months of age. Subsequently, her parents noticed that our patient did not respond to their calls and seemed isolated with introverted behavior. Our patient was not communicative and was consistently unresponsive to the calling of her name. In addition, she did not interact with other children and presented with restricted interest and repetitive stereotypical behavior. Our patient did not show imitative or projective play. In terms of education, starting at two years of age she was schooled with additional support from an early learning program. Brain magnetic resonance imaging (MRI) scans appeared normal. Our patient was diagnosed with pervasive developmental disorder of the autistic spectrum and moderate mental retardation.

**Table 1:** Pediatric environmental history can be used to identify the absence or presence of risk factors associated with autism spectrum disorder

Category	Factor
Constitutional and genealogical factors	Sex
	Race/ethnicity
	Family history
	Family tree

Reproductive history	Pregnancies
	Hormonal therapy

Environmental factors	Socioeconomic status
	Home
	Community
	Medical history of ionizing radiation
	Legal (alcohol and tobacco) and illegal drug use
	Pharmaceuticals/medications
	Occupational exposures
	Hobbies
	Home remedy/herbal supplement use

The 32-year-old mother and 36-year-old father lived on the second floor of a 14-year-old building in Puente Tocinos, Murcia. The mother had given birth to a healthy boy six years earlier. In the current case, the pregnancy was not planned. During the peri-conceptional stage, the mother ingested approximately 20 to 40 g of ethanol per day, while the father ingested around 40 to 60 g of ethanol per day during the first nine days of embryonic development. The alcohol intake subsequently decreased to 10 g/day for both parents until the 16th to 18th days of embryonic development when the parents became aware of the pregnancy and ethanol consumption was completely eliminated. Neither parent smoked during the pregnancy or during our patient's first year of life, and our patient was not exposed to any other drugs. Her serology was normal, and blood test results were positive for rubella IgG antibodies. Both parents had post-high-school education.

The mother was employed in the food manufacturing industry and occupational exposures during pregnancy included: phosphoric acid, alkylbenzenesulfonic acid, hydroxide and sodium hypochlorite, nitric acid, sodium hydroxide and alkyl alcohol ethoxylate. The father was employed as an auto mechanic and reported a null or slight possibility that he had indirectly brought home traces of chemicals or solvents that had soiled his clothing or shoes.

From a year prior to conception the mother began a weight loss diet and ingested approximately 1200 mg/day of 'horsetail' (*Equisetum arvense*) herbal remedies up to three years after birth. At conception maternal body mass index (BMI) was 31.6 kg/m^2^, which decreased to 30.1 kg/m^2 ^at the end of gestation, a net loss of almost 4 kg. Throughout pregnancy, despite adequate caloric intake, the mother reported a daily food intake of folate (199 μg), vitamin B1 (1.18 mg), vitamin B6 (1.31 mg) and vitamin B12 (30.8 μg). Vitamin B12 and folate intake supplements were started on approximately days 42 to 48 of gestation.

## Discussion

In general, a careful PEH requires basic knowledge and instruction in order to orient and guide the anamnesis towards risk factors of the disease. An adequate training in PEH includes not only the theoretical aspects (such as knowing the related RFs), but also the ability to characterize and quantify exposures while communicating the pertinent risks and helping to avoid unnecessary alarm and/or omissions of relevant clinical information. This is especially important in cases such as ours, when a disease such as the autism spectrum was documented and may have been associated with exposures that had a high degree of uncertainty [6].

Initially, we conducted an extensive review of the literature published since 1985 in PubMed Clinical Queries, Developmental and Reproductive Toxicology Database (DART) and Integrated Risk Assessment (IRIS). A selective search for chemicals in the Hazardous Substances Data Bank (HSDB) was also completed. The keywords used were: 'Autism Spectrum Disorder' OR 'Autism' AND 'Risk Factor'. There was insufficient evidence to associate any chemical exposure to the diagnosis of ASD, although there is some evidence suggesting that some prenatal factors increase the risk. Table 2 summarizes the environmental risk factors studied in the scientific literature that may increase the risk of developing the ASD.

**Table 2:** Environmental risk factors studied in autism/autism spectrum disorder (ASD) in the scientific literature

Risk factors	Comments
Thalidomide	Time of critical exposure: 20 to 24 days after conception

Misoprostol	Time of critical exposure: first trimester, near sixth week after conception. Used for illegal abortion.

Valproic acid	Time of critical exposure: first 3 to 4 weeks after conception. Neural tube defects, cardiac malformations, craniofacial malformation.

Rubella infection	Time of critical exposure: infection during the first 8 weeks

Chlorpyrifos	Pesticide used at home, school, community and farms

Organochlorated pesticides	Dicofol and endosulfan exposure. First to eighth weeks. Correlation between maternal residence near agricultural pesticide exposure and autism.

Prenatal, neonatal and perinatal factors	Advanced maternal and/or paternal age (mother > 35; father > 40); bleeding during pregnancy; forceps or vacuum delivery; prolonged labor; low birth weight (< 2500 g); respiratory distress syndrome; meconium aspiration syndrome; preterm birth at < 33 weeks; breech presentation; gestational age < 35 weeks; mothers who used medicine during pregnancy

Maternal immigration/mother born abroad	Increased risk of ASD according to region and ethnicity; more risk in Caribbean and African-American populations

Daily smoking in early pregnancy	The risk of autism is associated with daily smoking in early pregnancy

β2-Adrenergic receptor agonist	Used to treat premature labor. Continuous terbutaline exposure for 2 weeks had increased risk for ASD.

Birth defects	Associated with a near twofold increased risk for autism overall

Chlorinated solvents and heavy metals	Association between autism and estimated concentrations in ambient air around birth residence. Increased risk for solvent and metals (mercury, cadmium, nickel, trichloroethylene and vinyl chloride).

Parental psychiatric history	Parental psychopathology is associated with risk of autism and effective disorders

Alcohol and drugs	It is very unlikely that there is a strong association between prenatal alcohol exposure and autism

High parental education	Families with higher education background will seek services, thus reporting a child with autism

Lack of omega 3 fatty acids	Studies showed link between childhood development disorders and omega-6, omega-3 imbalances

Congenital cytomegalovirus (CMV) infection	Timing of injury to the developing brain by CMV may be in the third trimester in some patients with ASD

Singleton and concordant multiple births	Results indicated that ASD-concordant multiple births in boys tended to be higher than expected in March, May and September, but were 87% less in December, as compared with January

Maternal autoimmune disorders	Maternal autoimmune disorders in women around the time of pregnancy are unlikely to contribute significantly to risk of autism (case-control study)

Fetal alcohol syndrome (FAS) (case report)	Autistic behavior has not been previously associated with FAS. No statistical data, however it raises awareness that FAS could be a risk factor that should be evaluated by physicians.

Neonatal hyperbilirubinemia	Not a risk factor associated with ASD. Children with any degree of bilirubin level elevation were not at increased risk of ASD.

Antenatal ultrasound	Antenatal ultrasound is unlikely to increase the risk of ASD (case-control study)

Ammonium perchlorate	No reports of risk found

Mercury (vaccines)	No risk found

Measles, mumps, rubella (MMR) vaccination	No evidence that supports MMR vaccination relationship with autism

The causes for developmental disorders such as ASD are currently unknown. Genetic factors may provide some explanation. However, there is overwhelming evidence that environmental exposures during the period prior to conception and gestational weeks two to six are critical periods. Unfortunately, these are the periods of time when, in the majority cases, the pregnancies are still unrecognized by the prospective parents.

Of importance in this case, maternal ethanol consumption continued until early gastrulation although physical features of fetal alcohol syndrome were not observed. Alcohol is a very potent toxin to the fetal nervous system and the relationship with autism remains controversial. It has been reported that up to 9% of children born to mothers that consume alcohol during pregnancy are autistic [7]. However, autistic symptoms *per se *are usually not considered to be part of fetal alcohol syndrome. However, recent case-control studies suggest that prenatal alcohol exposure does not increase the risk of ASD [8]. Therefore, a strong association between prenatal alcohol exposure and autism is highly unlikely.

Autism is common in populations exposed to a teratogenic insult around the time of gastrulation [9]. It is now established that children with fetal alcohol spectrum disorders (FASDs) display deficits in executive functioning (EF). Despite marked differences in their clinical presentation, children with autism also demonstrate pronounced deficits in EF. Therefore, it is reasonable to ask the question: is the pattern of executive deficits in FASDs different from that in autism?

Occupational exposures of the mother and father of our patient were revised in DART and HSDB, but we found no relation with ASD and exposure effects during neurodevelopment.

Horsetail remedies are not recommended for use during pregnancy or breastfeeding, since little information is available on their safety. Horsetail remedies have been known to cause neurodevelopmental toxicity, and have a high potential to cause thiamine depletion and nicotine-like effects [9-11]. Thiamine is water soluble and has a short half-life. Thiamine status can be altered due to: dietary thiamine deficiency, breakdown by thiaminase and the administration of thiamine analogues [12]. Recent phytochemical analyses have detected the presence of tannins, saponins, sterols and flavonoids in horsetail residues [13-15]. In addition, horsetail contains thiaminase, an enzyme that destroys thiamine (vitamin B1) and, with long-term use, could lead to vitamin deficiency [11]. Thiaminase-induced deficiency of thiamine has been implicated to thiamine degradation by thermolabile thiaminases present in raw fish and shellfish [16].

Plant thiamine antagonists are heat stable and occur as both orthohydroxyphenols and parahydroxyphenols. Some examples of these antagonists are caffeic acid, chlorogenic acid, and tannic acid. These compounds interact with thiamine to oxidize the thiazole ring, thus rendering it unable to be absorbed resulting in thiamine deficiency. Two flavonoids, quercetin and rutin, have also been implicated as thiamine antagonists [17]. Several spontaneous central nervous system disorders due to thiaminase effects have occurred in experimental animal studies [18].

Horsetail remedies also contain diuretic properties used for weight control, which may explain our patient's mother's weight loss despite adequate caloric intake, an unusual occurrence in pregnant women. In our patient's mother, the ingestion of B-complex vitamins did not reach the recommended daily allowance levels for pregnant women (thiamine 1.4 mg/day, folic acid 600 μg/day) and lactating women (thiamine 1.4 mg/day, folic acid 500 μg/day). Additionally, possible neurotoxicity caused by thiamine and folic acid deficiency could have been potentiated by the horsetail's anti-thiamine activity and ethanol exposure during early pregnancy [11,18-20].

We believe that PEH is the best clinical tool to approximate the etiology of multi-factorial pediatric diseases. PEH is the basic and most essential work tool of Pediatric Environmental Health Specialty Units. However, the individual risk assessment for these patients is a complex process that requires specific diagnostic abilities. Pharmacological use during pregnancy is a known risk factor for autism and the relationship described seems plausible, however it is necessary to be cautious with the interpretation of these issues [21]. Although the use of herbal supplements during pregnancy is relatively common, there is little information on their effects on fetal development. This work also serves to highlight potential secondary physiological effects of over-the-counter herbal remedies, which may include micronutrient imbalance. This case report has several limitations such as the inconsistent composition of herbal supplements, possible interaction between multiple exposures and the lack of biomarkers in the study. However, the case serves to illustrate a possible mechanism of developmental neurotoxicity through a vitamin deficiency potentiated by herbal supplementation and alcohol exposure.

## Conclusions

As further scientific evidence accumulates, a detailed and careful PEH should be obtained from all patients with ASD. After analyzing this particular case, it is recommended that maternal exposure to ethanol and herbal supplements with suspected or potential toxicity should be avoided during pregnancy. Prospective parents should perform preconception planning before becoming pregnant.

## Consent

Written informed consent was obtained from the patient's next-of-kin for publication of this case report and any accompanying images. A copy of the written consent is available for review by the Editor-in-Chief of this journal.

## Competing interests

The authors declare that they have no competing interests.

## Authors' contributions

JAOG, MGA and ES conducted the pediatric environmental history. JAOG, ES, MGA, APM, EMS, LC and OPS analyzed and interpreted data from our patient regarding the disease. All authors read and approved the final manuscript.
